# Cardiovascular Risk Factors before Onset of Rheumatoid Arthritis Are Associated with Cardiovascular Events after Disease Onset: A Case–Control Study

**DOI:** 10.3390/jcm11216535

**Published:** 2022-11-03

**Authors:** Heidi Kokkonen, Linda Johansson, Hans Stenlund, Solbritt Rantapää-Dahlqvist

**Affiliations:** 1 Department of Public Health and Clinical Medicine, Rheumatology, Umeå University, 901 87 Umeå, Sweden; 2 Department of Public Health and Clinical Medicine, Epidemiology and Global Health, Umeå University, 901 87 Umeå, Sweden

**Keywords:** rheumatoid arthritis, cardiovascular disease, risk factors

## Abstract

Background: The increased comorbidity and mortality in rheumatoid arthritis (RA) patients are largely due to cardiovascular disease (CVD). Previously, we demonstrated increased frequencies of risk factors for CVD (elevated body mass index (BMI), elevated apoliprotein (Apo) B:ApoA1 ratio, and smoking) in pre-RA individuals compared with matched controls. Objectives: Assess the impact of traditional CV risk factors present before the onset of RA on the risk of CV events (CVE) after diagnosis in comparison with matched controls. Methods: A case–control study including 521 pre-symptomatic individuals and 1566 controls identified within the Health Surveys of the Medical Biobank was performed. CVD risk factors were hypertension, elevated ApoB:A1 ratio, BMI, diabetes, and smoking. Information on comorbidities was requested from the Swedish National Patient Register and Cause of Death Register. Results: Pre-RA individuals had a higher risk of future CVE compared with matched controls (HR [95% CI] 1.70 [1.31–2.21]), which remained after adjustments for risk factors for CVD (HR [95% CI] 1.73 [1.27–2.35]). Most risk factors were associated with CVE after diagnosis, and a combination resulted in a higher risk in RA compared with controls; two risk factors, HR [95% CI] 2.70 [1.19–6.13] vs. 1.26 [0.75–2.13]; and three to four risk factors, HR [95% CI] 6.32 [2.92–13.68] vs. 3.77 [2.34–6.00]. Conclusions: Risk factors for CVD present in pre-RA individuals were associated with future CVE, and even after adjustments for these risk factors and treatments after RA onset, pre-RA individuals had a higher risk of CVE compared with controls. These findings further highlight the importance of the early assessment of risk for CVD.

## 1. Introduction

Rheumatoid arthritis (RA) is a progressive systemic inflammatory disease characterized by joint destruction and functional disability. The increased comorbidity and premature mortality observed in patients with RA are largely due to cardiovascular disease (CVD) [1,2,3,4,5,6]. Meta-analyses have calculated that the risk of CV mortality is increased up to 60% in patients with RA [6]. The etiology of this increased morbidity and mortality due to CVD in patients with RA compared with the general population is not fully understood, although traditional risk factors such as smoking, hypertension, and diabetes as well as the inflammatory burden have been suggested [5,7,8]. We have previously shown that individuals who subsequently develop RA already have increased frequencies of several risk factors for CVD, such as elevated BMI, elevated apoliprotein (Apo) B:ApoA1 ratio, and cigarette smoking, compared with matched controls, before RA symptom onset [9].

A retrospective study showed that prior to RA diagnosis, patients had significantly higher risk for hospitalization for either acute or unrecognized myocardial infarction (MI) [3]. Recently, a retrospective case–control study analyzed the incidence of CVD in individuals prior to RA and after diagnosis, showing an excess of both stroke and heart failure prior to the diagnosis of RA [10]. Additionally, after diagnosis, an increased risk of CVD was seen that could not be fully explained by traditional CVD risk factors or RA-related factors at diagnosis [10].

Conflicting results have been presented regarding the association of CVD with markers of disease activity and severity in patients with RA: some studies have presented significant associations [5,7,11], whereas other studies have not been able to confirm this [12,13].

To our knowledge, no previous study has analyzed the association of risk factors already present prior to symptoms of RA in relation to CVE diagnosed after disease onset. In this study, with data collected before symptom onset from individuals who subsequently developed RA, we aimed to assess the impact of CV risk factors and their association with future CVE in comparison with matched controls. The disease activity and risk factors after RA onset were taken into account.

## 2. Study Cohorts and Methods

Information regarding the presence of the selected risk factors for CVD was based on surveys with data collected in the Västerbotten Intervention Program (VIP) and the Northern Sweden Multinational Monitoring of Trends and Determinants in Cardiovascular Disease (MONICA) as previously described [9,14,15]. VIP is a population-based study aimed at reducing morbidity and mortality due to CVD and diabetes in northern Sweden. The procedures for participation, blood sampling, and collection of anthropometric data have been described previously [9,14].

To identify individuals who had participated in VIP or MONICA and subsequently developed RA, a co-analysis of these registers with the registers of patients fulfilling the 1987 American Rheumatism Association [16] criteria for RA (since 1 January 1996) at the department of Rheumatology, University Hospital, Umeå, Sweden, was performed in 2014. In total, 547 individuals were identified as having participated in VIP or MONICA surveys prior to the onset of symptoms of joint disease, that is, “pre-RA or pre-symptomatic individuals”. For each of them, three controls were selected from the same registers, matched for year of birth and participation in VIP/MONICA, sex, and living conditions (rural or urban). A total of 1641 controls, who until the linkage was performed did not have a diagnosis of RA, were identified. For the present study, 521 of the pre-symptomatic cases and 1566 controls had available and usable data.

Using patients’ unique personal identity numbers, we requested information on comorbidities, coded according to the International Statistical Classification of Diseases and Related Health Problems 10th revision (ICD-10), or in a few individuals according to ICD-9, from the Swedish National Patient Register. This register has data on inpatient care, with full coverage since 1987. Since 2001, data from outpatient care, except for primary health care, have also been included. Cause of mortality data were available from the Cause of Death Register, with data since 1961. In the present study, information on comorbidities and cause of mortality was available until the end of the study period on 31 December 2016. The selected ICD-10 codes for comorbidities or mortality due to CVE are presented in Supplementary Table S1 and a flow-chart of the procedure of inclusion in the study is presented in Supplementary Figure S1.

The selected risk factors for CVD that were extracted from the health surveys and blood sampling of VIP/MONICA from the time pre-dating symptoms of RA were hypertension (systolic blood pressure (SBP) ≥140 mmHg and/or diastolic blood pressure (DBP) ≥90 mmHg, including hypertensive treatment), diabetes (self-reported in the questionnaires in VIP or MONICA), BMI (increased ≥25.0 kg/m^2^), ApoB:A1 ratio (elevated, defined as follows: women ≥0.7, men ≥0.8, including lipid-lowering therapy), and smoking (ever or current). These were selected according to earlier studies, as previously described [9]. A risk factor profile was constructed and defined by the number of risk factors present, with 0–1 as the reference and subjects subsequently grouped as having 2 or 3–4, as modified by Berry et al. [17].

In this study, a primary analysis was performed to explore if individuals (pre-RA [*n* = 521]) and controls (*n* = 1566) had developed a CVE prior to the date of diagnosis of RA (the index date) in pre-RA individuals or matched controls. If so, these individuals were excluded from further analyses. In total, 27 pre-RA cases and 97 controls had a CVE prior to the index date and thus were excluded, resulting in 494 cases and 1469 controls available for further analyses. The primary end points in the subsequent statistical analyses were the first diagnosis of a CVE, death, or the end of the study in individuals without CVE (31 December 2016) after RA onset. The date of diagnosis of RA in the cases was set as the index date for the cases and their matched controls.

Information regarding treatment for hypertension, lipid-lowering treatment, or diabetes mellitus after the date of diagnosis (index date) was collected for the patients from their medical records and used to make adjustments in the analyses. For the controls, no data regarding additional treatment after the index date were available. These controls were included from the population-based surveys performed at the Medical Biobank of Northern Sweden (VIP and MONICA), and data on the presence of CV risk factors and treatment for hypertension or lipid lowering treatment is only available from the sampling date and there is no collection of data on the general population as follow-up of the health surveys.

The frequency of different treatments for the patients during the first 24 months was analyzed and included conventional disease-modifying antirheumatic drugs (csDMARDs: azathioprine, chloroquine, cyclosporine, leflunomide, methotrexate, myocrisin, and sulfasalazine), biological disease-modifying antirheumatic drugs (bDMARDs: abatacept, adalimumab, anakinra, certolizumabpegol, etanercept, golimumab, infliximab, rituximab, and tocilizumab), and corticosteroids.

### 2.1. Measures of Disease Activity and Outcome

The disease activity score for 28 joints (DAS28) was calculated at baseline and every 6 months up to 24 months. This score included the number of tender and swollen joints, the patient’s score on a global-health visual analogue scale (VAS), and the erythrocyte sedimentation rate (ESR) [18]. The area under the curve for the first 24 months (AUC_24_) was calculated utilizing the DAS28 values for the different time points: baseline, 6, 12, 18, and 24 months [19].

### 2.2. Analyses of Apolipoproteins (ApoA1 and ApoB) and Autoantibodies

ApoA1 (g/L) and ApoB (g/L) were analyzed from stored samples from the Medical Biobank using an immunoturbidimetric method (Cobas 8000 instrument; Roche Diagnostics Scandinavia AB). Anti-citrullinated protein antibodies (ACPA) were analyzed using the anti-CCP2 enzyme-linked immunoassays (Euro-Diagnostica AB, Malmö, Sweden) both before symptom onset and at the index date, with a cut-off for positivity at 25 AU/mL. Rheumatoid factor (RF) was analyzed according to a routine clinical protocol (Waaler-Rose hemagglutination test).

### 2.3. Statistical Analyses

Statistics were performed using SPSS (IBM SPSS Statistics for Macintosh, version 26.0. Armonk, NY: IBM Corp, USA). To analyze the associations with risk of future RA, conditional logistic regression models were used, with calculated odds ratios (ORs) and 95% confidence intervals (CIs). Univariable and multivariable logistic regression analyses adjusted for age and sex were used to detect associations of different factors with CVE in pre-symptomatic individuals and controls, respectively. Cox proportional-hazard regression was used when time was taken into account. All *p*-values are two-sided and *p* < 0.05 was considered statistically significant.

Data on BMI, blood pressure, smoking (ever/never and current/previous), and diabetes were available for 97.7–99.5% of the individuals, whereas results for ApoA1 and ApoB levels were available for 83% of the cases and 85% of the controls. Imputation for missing data for the ApoB:ApoA1 ratio was constructed using logistic regression based on CV risk factors, sex, and age. Sensitivity analyses after imputation of data for the ratio of ApoB to ApoA1 (15.3% among controls and 17.1% in the pre-symptomatic individuals, respectively) did not differ in CV risk factors, future CVE, and age and sex distribution.

## 3. Results

In this longitudinal case–control study, the association of risk factors for CVD identified in individuals prior to the onset of symptoms of joint disease were analyzed for future development of CVE after RA onset in comparison with matched controls. The risk of CVE was adjusted for RA-related factors and additional risk factors developed after onset as covariates.

Demographic and clinical data of the included individuals prior to and at RA diagnosis, and frequencies of CVE, respectively, are presented in Table 1. The median time to onset of symptoms from the date of collection of data and samples among the pre-symptomatic cases was 5.7 years (IQR 6.5 years) (Table 1). The total time at risk for a CVE during the study period was 5116 person-years, with an incidence rate of a first CVE of 16.8/1000 person-years for the cases, and 16,097 person-years with an incidence rate of 10.1/1000 person-years for the controls.

### 3.1. CVD Risk Factors in Relation to CVE in Pre-Symptomatic Individuals

Including individuals who already had a CVE prior to the index date (i.e., the date of a diagnosis of RA for the cases and their corresponding matched controls), 21.7% had at least one registered CVE compared with 16.7% among the matched controls (Table 1, OR [95% CI] 1.43 [1.10–1.84], *p* = 0.007). After excluding individuals who had a CVE prior to the index date (*n* = 27 cases and *n* = 97 controls, respectively), the corresponding frequency was significantly higher for CVE in cases compared with controls: 16.5% vs. 10.4% (Table 1, OR [95% CI] = 1.93 [1.19–3.14], *p* = 0.008).

Univariable Cox proportional-hazard regression models taking time into account and treating CVE after disease onset as the end point showed that all the included risk factors identified during the time before disease onset were significantly associated with a future CVE (Table 2). After adjustment for the various risk factors for CVD in a multivariable model, pre-symptomatic individuals had a significantly higher risk of future CVE compared with matched controls (HR [95% CI] = 1.66 [1.22–2.26], *p* = 0.001, Table 2).

To compare the impacts of the various risk factors for CVD between the pre-symptomatic individuals (prior to symptoms of RA) and controls, stratified analyses were performed for the two groups. Both in pre-symptomatic individuals and controls, current smoking, hypertension, and an elevated ApoB:ApoA1 ratio were significantly associated with a future CVE (Table 3), whereas an elevated BMI was significant only in the controls (Table 3). Including all the CVD risk factors in a multivariable analysis showed that all factors that were significant in the univariable analyses remained significant for CVE in the pre-symptomatic cases while, in the controls, all variables except BMI remained significant, whereas only ApoA/apoB remained significant in pre-symptomatic cases when including only significant factors from univariable analyses (Table 3).

### 3.2. CV Risk Factors in Relation to CVE in Pre-Symptomatic Individuals and Controls

A composite risk variable consisting of a combination of the most frequent risk factors for CVD (current smoking, BMI ≥ 25.0, elevated ApoB:ApoA1 ratio, and hypertension) was constructed to analyze the impact on future CVE of having several CVD risk factors compared to none or one prior to onset of RA. These combinations showed that having two, or three to four, CVD risk factors already prior to a diagnosis of RA was associated with a significantly higher risk for future CVE in the cases, while in the matched controls the risk for future CVE was significant only with three to four risk factors compared with having none to one (Table 4).

A summary of combinations of the significant risk factors present prior to the index date (hypertension, elevated ApoB:A1 ratio, and current smoking) are presented in Supplementary Table S2. The combinations resulted in similar HRs in the subsequent RA cases and the matched controls. Particularly, the presence of hypertension seemed to have the highest impact in the combinations in both groups, although, the 95% CIs are wide and overlapping. Elevated ApoB:ApoA1 ratio in combination with hypertension yielded a higher OR compared with the combination of hypertension with current smoking in the cases, while in controls these risk factor combinations acted to the opposite effect.

### 3.3. Cardiovascular Risk Factors and Clinical Data in Relation to CVE in RA after Index Date

For the patients, information regarding additional treatment (i.e., treatments prescribed after diagnosis of RA) for hypertension and diabetes mellitus, and lipid-lowering treatment, after the index date were used for adjustments in the statistical analyses. The number of additional treatments after disease onset were as follows: for hypertension, 111/470 (23.6%); for lipid-lowering treatment, 34/428 (7.9%); and for diabetes, 18/374 (4.8%); through the end of the study. Analyses with the same variables as in Table 3 were performed with adjustments for additional treatments after the index date and showed that all the significant associations remained (Table 5).

Disease activity in the patients as measured with DAS28 at the index date or during the first 24 months were not significantly associated with CVE (Table 5). Having treatment with a biological DMARD (bDMARD) showed a borderline association with CVE in cases in univariable models (HR [95% CI] = 1.74 [0.99–3.06], *p* = 0.053). In multivariable analyses including all significant factors from the univariable analyses, bDMARD use was associated with a CVE (HR [95% CI] = 2.63 [1.27–5.41], *p* = 0.009). This was significant in addition to the other risk factors (present prior to the index date) in the multivariable model, including elevated ApoB:ApoA1 ratio (HR [95% CI] = 5.08 [2.37–10.87], *p* = 2.90 × 10^−5^), current smoking (HR [95% CI] = 1.99 [1.19–3.34], *p* = 0.009), and hypertension (HR [95% CI] = 3.50 [1.80–6.81], *p* = 2.16 × 10^−4^) (Table 5). There were no significant associations of corticosteroids or csDMARDs to CVE (data not shown). Patients with bDMARD had significantly higher DAS28-AUC_24_ compared with patients without bDMARD (OR [95% CI] = 1.03 [1.02–1.05], *p* = 4.8 × 10^−5^, adjusted for age and sex.

Analyzing associations of the presence of ACPA per se prior to the onset of symptoms of RA or at the index date (i.e., at diagnosis) revealed no significant associations with the development of future CVE in the patients with RA (*p* = 0.44, and *p* = 0.57, respectively, Table 5), nor did the presence of RF at the index date show any significant associations with future CVE (*p* = 0.77, Table 5). When stratifying the patients according to autoantibody status at the index date, analyses showed an increased risk of future CVE compared with that of the controls for the ACPA-positive patients (i.e., ACPA positive at diagnosis) (HR = 1.82 [95% CI 1.34–2.45], *p* = 1.04 × 10^−4^) no association was seen for the ACPA-negative cases (HR = 1.40 [95% CI 0.80–2.48], *p* = 0.24). Stratification according to RF status showed significant associations with future CVE irrespective of positivity (HR = 1.69 [95% CI 1.27–2.25], *p* = 3.16 × 10^−4^) or negativity (HR = 1.82 [95% CI 1.11–2.87], *p* = 0.014).

## 4. Discussion

We have shown that known risk factors for CVD already present in individuals prior to RA symptom onset were associated with future CVE. Even after adjustment for the risk factors for CVD after disease onset, those individuals who later developed RA had a higher risk of future CVE in comparison with the matched controls. 

Additionally, a combination of increasing numbers of risk factors present before symptom onset was associated with increasing risk of future CVE, in both cases and controls. In cases, having two pre-existing risk factors was associated with an increased risk compared with having none or one, and this was not seen among controls. 

Despite these risk factors being present prior to the onset of RA, there were no significant differences in the frequency of CVE in individuals before RA onset compared with controls, which could indicate that the increased risk of CVE in patients with subsequent RA appears after RA onset, together with increased inflammatory activity. Of those individuals who later was diagnosed with RA but did not yet have symptoms of RA, 36% were ACPA positive, which could suggest that in these individuals the progression towards RA already has started. However, the fact that the number of individuals with an event before the onset of RA was low should be taken into account. In a study by Nikiphorou et al., an increased frequency of stroke and heart failure was already present prior to the diagnosis of RA [10]. We chose not to include heart failure in the diagnoses of CVE, as we suspect that this diagnosis could occasionally be applied to vague findings, such as a slight increase of NT-pro brain natriuretic peptide that could be due to other causes.

Analyzing traditional risk factors for CVD that were present prior to the index date (i.e., before RA diagnosis) for risk of future CVE showed that all analyzed factors except for diabetes were significantly associated with future CVE in multivariable analyses. The lack of association of diabetes with CVE in this study could have occurred for several reasons. For example, the diagnosis of diabetes prior to RA as well as medical treatment for diabetes was self-reported in the questionnaires, which could lead to an under-reporting of this diagnosis. Only 1.4% of the controls and 3.3% of the patients reported that they had diabetes, and stratification rendered too few cases with CVE. Additionally, the survey participants were 40, 50 and 60 years old, and especially type II diabetes mellitus is usually diagnosed at an older age.

In multivariable analyses including significant factors from the univariable analyses, only elevated ApoB:ApoA1 ratio remained significant in the patients. However, in controls, hypertension, current smoking and elevated ApoB:ApoA1 ratio were significant in multivariable models, whilst increased BMI did not remain significant.

Previous studies have shown an increased risk of comorbidities in patients with higher inflammatory activity measured with ESR and DAS28-AUC_24_ [5,20,21]. Here, we could not demonstrate a significant association of disease activity at baseline or during the first 2 years with CVE. However, treatment with a bDMARD was associated with CVE, and this group had significantly higher DAS28-AUC_24_ compared with those without bDMARD treatment.

We were unable to demonstrate any associations of the presence of autoantibodies per se. Neither ACPA prior to onset of RA nor at the index date increased the risk of subsequent CVE. However, when stratifying the patients at the index date based on the presence of autoantibodies, the risk of future CVE among the RA patients compared with controls showed that an association with increased risk for CVE was restricted to ACPA-positive cases at the index date. The RF status at baseline did not affect the risk of CVE, and the association with CVE was similar irrespective of RF.

An increased risk of CV morbidity and mortality in autoantibody-positive patients compared with autoantibody-negative patients has been reported [22,23,24,25]. These studies have mainly been performed on established RA cohorts with longer disease duration, while our primary focus is on the pre-symptomatic phase and the first years after disease onset, although there are also studies in line with our results. Kerola and colleagues analyzed CV comorbidity at the time of diagnosis of RA and did not find any association of RF positivity with CVD [26]. In a study analyzing the risk of myocardial infarction in relation to RA, no significant differences were detected in the risk of hospitalization in RF-positive patients compared with RF-negative patients [27]. The association of an increased risk of future CVE mainly in the ACPA-positive patients at baseline could be due to this population’s higher inflammatory profile and could imply that ACPA-positive RA is a more severe disease, with potentially more comorbidity such as CVD, than ACPA-negative disease, as has been suggested by others [25]. We found that ACPA-positive patients had significantly higher disease activity (DAS28-AUC_24_) compared with ACPA-negative patients.

The strength of using data from the population-based cohorts of VIP and MONICA for a case–control study is that these well-defined cohorts incorporate individuals representing the population of Västerbotten who have already donated blood samples and completed questionnaires prior to the onset of symptoms of RA. This enables us to use matched controls from the same cohorts sampled and collected similarly. The diagnosis of RA is determined at the rheumatology department through clinical examination by a rheumatologist. The co-analysis with the Biobank was performed in 2014, and until then none of the controls had a diagnosis of RA.

A limitation of this study is the small number of individuals in each of the stratified subgroups, which prevented us from performing analyses such as calculating the risk for subgroups of CVEs, so we instead chose to analyze them as one group. For most of the risk factors for CVD prior to the onset of RA we had data for more than 97.0% of the individuals, and sensitivity analyses of data after multiple imputations did not show any significant differences in the data before and after imputation. Another limitation is that this is a retrospective study, and also there is always a risk in error of coding diagnosis according to the ICD-system used in the clinical setting. Furthermore, data regarding additional treatment after the index date were not available for the controls. Thus, we cannot ascertain how this may have affected the outcome of CVE. In patients with RA, we were able to collect information regarding treatment for hypertension and diabetes mellitus, as well as lipid-lowering treatment, and were able to adjust the calculations if the patients had received additional treatments for these conditions after the index date. Even after adjustments for these treatments after the index date, the significant associations of the pre-existing risk factors with future CVEs remained. Additionally, the use of non-steroid anti-inflammatory drugs (NSAIDs) can affect the risk for CVE, but we did only have information if cases had a prescription at the index date but no information on how or if they used these drugs. It is also a possibility that individuals buy NSAIDs without a prescription. So, due to these limited data, we chose not to include the information about NSAIDs in the analyses.

From this study, we can conclude that the presence of CVD risk factors before symptom onset was associated with an increased risk of a CVE after disease onset, a risk that remained even after adjustments for risk factors for CVD that were already present prior to symptoms of RA. Together, these results further highlight the importance of the early assessment of the risk of CVE by identifying CVD risk factors in individuals as soon as they are presented with a diagnosis of RA. They also show the importance of tight control of treatment in early disease to try to suppress disease activity and inflammation in order to decrease the risk for CVE. Still, more studies need to be performed to elucidate other factors contributing to the risk of future CVD in these individuals.

## Figures and Tables

**Table 1:** Descriptive information on pre-RA cases and control individuals identified from the interventional surveys.

	Cases (*n* = 521)	Controls (*n* = 1566)
**Female sex, *n* (%)**	354 (67.9)	1065 (68.0)
***At Interventional survey and biobank sampling:***		
**Age at sampling (median, IQ1–3)**	50.2 (47.9–60.0)	50.3 (47.8–60.0)
**Time to onset of symptoms (years, median, IQ1–3)**	5.7 (2.9–9.4)	-
**Time from symptom onset to diagnosis (months, median, IQ1–3)**	7.0 (4.0–12.0)	-
**ACPA ^a^ positivity before symptom onset, *n* (%)**	163/453 (36.0)	-
**HLA-SE, *n* (%)**	326/505 (64.6)	-
**Elevated ApoB:ApoA1 ratio ^b^, *n* (%)**	229/432 (53.0) *	635/1327 (47.9)
**Elevated BMI ^c^, *n* (%)**	314/518 (60.6) **	830/1559 (53.2)
**Hypertension ^d^, *n* (%)**	207/519 (39.9)	637/1548 (41.1)
**Ever smoker, *n* (%)**	353/518 (68.1) ***	794/1536 (51.7)
**Current smoker, *n* (%)**	185/512 (36.1) ***	313/1530 (20.5)
**Diabetes ^e^*, n* (%)**	17/518 (3.3) *	25/1558 (1.6)
**Hypertensive treatment ^e^**	73/431 (16.9)	232/1271 (18.3)
**Lipid-lowering treatment ^e^**	13/372 (3.5)	46/1111 (4.1)
***At diagnosis of RA:***		
**Age at diagnosis (median, IQ1–3)**	60.8 (52.2–67.4)	-
**ACPA positivity at diagnosis, *n* (%)**	362/448 (80.8)	-
**RF positivity at diagnosis, *n* (%)**	407/517 (78.7)	-
**NSAID ^f^ prescription, *n* (%)**	391/474 (82.5)	
***Treatment of RA during first 24 months:*** **Corticosteroids, *n* (%)** 311/467 (66.6) ***csDMARD ^g^, n* (%)** 450/479 (93.9) ***bDMARD ^h^, n* (%)** 84/481 (17.5) ***Cardiovascular event (CVE):***		
**CVE ever, *n* (%)**	113/521 (21.7) **	260/1566 (16.6)
**CVE after index date ^i^**	86/521 (16.5) ***	163/1566 (10.4)
**CVE before index date ^i^**	27/521 (5.2)	97/1566 (6.2)

^a^ ACPA, anti-citrullinated protein antibodies. ^b^ Elevated apolipoprotein (Apo), ApoB:ApoA1 ratio ≥ 0.8 in males, ≥0.7 in females. ^c^ Elevated BMI, body mass index ≥ 25.0 kg/m^2^. ^d^ Systolic blood pressure (SBP) ≥ 140 mmHg and/or diastolic blood pressure (DBP) ≥ 90 mmHg, including hypertensive treatment. ^e^ Self-reported in questionnaires. ^f^ NSAID, non-steroid anti-inflammatory drug, ^g^ csDMARDs, conventional synthetic disease-modifying rheumatic drugs. ^h^ bDMARDS, biological disease-modifying rheumatic drugs, ^i^ Index date, time point of diagnosis of RA for each RA patient and their matched controls. * *p* < 0.05, ** *p* < 0.01, *** *p* < 0.001 in conditional logistic regression models.

**Table 2:** Impact of various risk factors identified before RA symptom onset for the development of a cardiovascular event after onset.

	Univariable HR (95% CI)	*p*-Value	Multivariable HR (95% CI)	*p*-Value
**Case/control**	1.65 (1.27–2.14)	1.72 × 10^−4^	1.66 (1.22–2.26)	0.001
**Hypertension ^a^**	2.04 (1.56–2.66)	1.5 × 10^−7^	1.98 (1.44–2.70)	2.0 × 10^−5^
**Current smoking**	1.74 (1.32–2.28)	6.8 × 10^−5^	1.72 (1.26–2.34)	0.001
**Ever smoking**	1.39 (1.07–1.81)	0.013	-	-
**Elevated apo ratio ^b^**	2.42 (1.76–3.33)	6.5 × 10^−8^	2.30 (1.64–3.22)	1.0 × 10^−6^
**BMI ≥ 25.0 ^c^**	1.38 (1.06–1.81)	0.018	1.11 (0.80–1.54)	0.54
**Diabetes ^d^**	2.09 (1.07–4.08)	0.030	2.42 (1.22–4.79)	0.011

Cox proportional-hazard regression analyses (univariable and multivariable, hazard ratio [HR] 95% confidence interval [CI]) for development of a cardiovascular event (CVE) with time to event (CVE or end of study), excluding those with a CVE before the index date (time point of diagnosis of RA for each RA patient and their respective matched controls). ^a^ Systolic blood pressure ≥ 140 and/or diastolic blood pressure ≥ 90 and/or medical treatment for hypertension. ^b^ Elevated apolipoprotein (Apo) ratio, ApoB:ApoA1 ratio ≥ 0.8 in males, ≥0.7 in females. ^c^ BMI, body mass index ≥ 25 kg/m^2^. ^d^ Self-reported in questionnaires.

**Table 3:** Association of risk factors for cardiovascular disease with cardiovascular events in pre-RA and control individuals.

	*Univariable Model*				Multivariable Model Including all * Traditional CVD Risk Factors				*Multivariable Model—* *Including Significant Factors from Univariable Analyses*			
	Pre-RA Individuals (*n* = 493)		Controls (*n* = 1468)		Pre-RA Individuals (*n* = 493)		Controls (*n* = 1468)		Pre-RA Individuals (*n* = 493)		Controls (*n* = 1468)	
	OR (95% CI)	*p*-Value	OR (95% CI)	*p*-Value	OR (95% CI)	*p*-Value	OR (95% CI)	*p*-Value	OR (95% CI)	*p*-Value	OR (95% CI)	*p*-Value
**Hypertension ^a^**	**2.88** **(1.74–4.78)**	**4.2** × **10^−5^**	**2.24** **(1.57–3.19)**	**8.0** × **10^−6^**	**3.18** **(1.53–6.62)**	**0.002**	**2.20** **(1.32–3.68)**	**0.003**	2.13 (0.91–5.00)	0.081	**2.26** **(1.35–3.76)**	**0.002**
**Ever smoking**	1.52 (0.87–2.66)	0.137	1.17 (0.84–1.65)	0.358	-	-	-	-	-	-	-	-
**Current smoking**	**1.96** **(1.13–3.59)**	**0.030**	**1.72** **(1.10–2.69)**	**0.017**	**2.52** **(1.22–5.22)**	**0.013**	**1.77** **(1.06–2.97)**	**0.029**	1.47 (0.52–4.12)	0.460	**1.78** **(1.06–2.97)**	**0.028**
**BMI ≥ 25.0 ^b^**	1.15 (0.69–1.90)	0.591	**1.43** **(1.01–2.04)**	**0.047**	0.77 (0.36–1.63)	0.494	1.31 (0.78–2.21)	0.310	-	-	1.35 (0.80–2.20)	0.262
**Elevated ApoB:ApoA1 ratio ^c^**	**2.85** **(1.60–5.08)**	**4.02** × **10^−4^**	**2.92** **(1.91–4.46)**	**6.96** × **10^−7^**	**3.23** **(1.47–7.06)**	**0.003**	**1.80** **(1.07–3.02)**	**0.027**	**3.26** **(1.05–10.17)**	**0.042**	**2.62** **(1.68–4.09)**	**0.025**
**Diabetes ^d^**	1.06 (0.32–3.52)	0.923	2.06 (0.72–5.85)	0.177	3.22 (0.59–17.61)	0.177	2.29 (0.63–8.28)	0.207	-	-	-	-

Odds ratio with 95% confidence interval (OR, 95% CI) for cardiovascular events after index date (time point of diagnosis of RA for each RA patient and their matched controls), adjusted for age and sex. ^a^ Hypertension, systolic blood pressure (SBP) ≥ 140 mmHg and/or diastolic blood pressure (DBP) ≥9 0 mmHg, including hypertensive treatment. ^b^ Elevated body mass index (BMI), BMI ≥ 25.0, kg/m^2^. ^c^ Elevated apolipoprotein (Apo)B:ApoA1 ratio, male sex ≥ 0.8, female sex ≥ 0.7. * Never smoking. ^d^ Self-reported in questionnaires.

**Table 4:** Risk for CVE ^a^ stratified on number of CVD ^b^ risk factors ^c^ in pre-RA subjects and controls.

No. Risk Factors	CVE No, *n* (%)	CVE Yes, *n* (%)	HR (95% CI)	*p*-Value
**Pre-RA Individuals**				
**0–1**	148 (43.3)	8 (11.9)	Ref	
**2**	107 (31.3)	21 (31.3)	**2.70 (1.19–6.13)**	**0.017**
**3–4**	87 (25.4)	38 (56.7)	**6.32 (2.92–13.68)**	**3.00 × 10^−6^**
**Controls**				
**0–1**	557 (51.1)	28 (23.7)	Ref	
**2**	339 (31.1)	29 (24.6)	1.26 (0.75–2.13)	0.383
**3–4**	193 (17.7)	61 (51.7)	**3.77 (2.34–6.00)**	**1.95 × 10^−8^**

Calculations performed with Cox proportional-hazard regression models presented with hazard ratio (HR) with 95% confidence interval (95% CI), adjusted for age and sex. ^a^ CVE, cardiovascular event. ^b^ CVD, cardiovascular disease. ^c^ Including elevated body mass index (BMI) ≥ 25.0 kg/m^2^, current smoking, elevated apolipoprotein (Apo)B:ApoA1 ratio (male sex ≥ 0.8, female sex ≥ 0.7) and/or lipid-lowering treatment, and hypertension (systolic blood pressure ≥ 140 and/or diastolic blood pressure ≥ 90 and/or medical treatment).

**Table 5:** Association of risk factors for cardiovascular disease (CV) with development of a future CV event (after the index date ^1^) in individuals prior to the onset of RA (*n* = 494), and disease related factors at index date, adjusted for age, sex, and additional treatments (for hypertension, lipid-lowering treatment, or for diabetes mellitus) after the index date.

	*Univariable Model*		*Multivariable Model—* Including All * Traditional CVD Risk Factors		*Multivariable Model—* *Including Significant Factors from Univariable Analyses*	
	HR (95% CI)	*p*-Value	HR (95% CI)	*p*-Value	HR (95% CI)	*p*-Value
**Hypertension ^a^**	**3.18** **(1.79–5.64)**	**7.7 × 10^−5^**	**3.50** **(1.67–7.34)**	**0.001**	**3.50** **(1.80–6.81)**	**2.16 × 10^−4^**
**Ever smoking**	1.52 (0.92–2.53)	0.104	-	-	-	-
**Current smoking**	**1.66** **(1.07–2.57)**	**0.023**	**2.32** **(1.24–4.31)**	**0.008**	**1.99** **(1.19–3.34)**	**0.009**
**BMI** **≥ 25.0 ^b^**	1.18 (0.75–1.85)	0.471	0.80 (0.40–1.63)	0.546	-	-
**Elevated ApoB:ApoA1 ratio ^c^**	**3.16** **(1.40–7.13)**	**0.006**	**5.08** **(2.19–11.79)**	**1.52 × 10^−4^**	**5.08** **(2.37–10.87)**	**2.90 × 10^−5^**
**Diabetes ^d^**	2.08 (0.74–5.84)	0.163	2.15 (0.69–6.71)	0.187	-	-
**DAS28 at baseline ^e^**	1.02 (0.83–1.25)	0.854			-	-
**DAS28-AUC_24_^f^**	1.01 (1.00–1.02)	0.132			-	-
**bDMARD ^g^**	1.74 (0.99–3.06)	0.053			**2.63** **(1.27–5.41)**	**0.009**
**ACPA ^h^**	1.20 (0.65–2.19)	0.565				
**RF ^i^**	1.09 (0.65–1.81)	0.770				

Hazard ratios with 95% confidence intervals (HR, 95% CI) for cardiovascular events after the ^1^ index date (time point of diagnosis of RA), adjusted for age and sex. ^a^ Hypertension (systolic blood pressure (SBP) ≥ 140 mmHg and/or diastolic blood pressure (DBP) ≥ 90 mmHg, including hypertensive treatment), adjusted for additional hypertension after the index date. ^b^ Elevated body mass index (BMI) BMI ≥ 25.0, kg/m^2^. ^c^ Elevated apolipoprotein (Apo)B:ApoA1 ratio: male sex ≥ 0.8, female sex ≥ 0.7, adjusted for additional lipid-lowering treatment after the index date. * Not ever smoking. ^d^ Self-reported in questionnaires, adjusted for diabetes mellitus after the index date. ^e^ Disease activity score for 28 joints. ^f^ DAS28 AUC for the first 24 months. ^g^ Biological disease-modifying anti-rheumatic drug, ^h^ ACPA, anti-citrullinated protein antibodies at index date, ^i^ RF, rheumatoid factor at index date.

## Data Availability

The data underlying this article cannot be shared publicly due to the privacy of individuals that participated in the study. The data will be shared on reasonable request to the corresponding author.

## Supplementary Materials

The following supporting information can be downloaded at: https://www.mdpi.com/article/10.3390/jcm11216535/s1, Figure S1: Flowchart on the inclusion of pre-RA individuals and matched controls in the study; Table S1: ICD^a^-10/-9 codes included as a CVE^b^ due to CV comorbidity or mortality; Table S2: Risk for CVE^a^ after index date stratified on CVD^b^ risk factors prior to RA onset.
