# Supplementary material for: Cardiovascular Risk Factors before Onset of Rheumatoid Arthritis Are Associated with Cardiovascular Events after Disease Onset: A Case–Control Study
Source: J Clin Med. 2022 Nov 3;11(21):6535. doi: 10.3390/jcm11216535 (PMC9658375; doi:10.3390/jcm11216535)
Supplement: Supplementary file 1 [file jcm-11-06535-s001.zip › Supplementary Figure S1.hk.pdf]

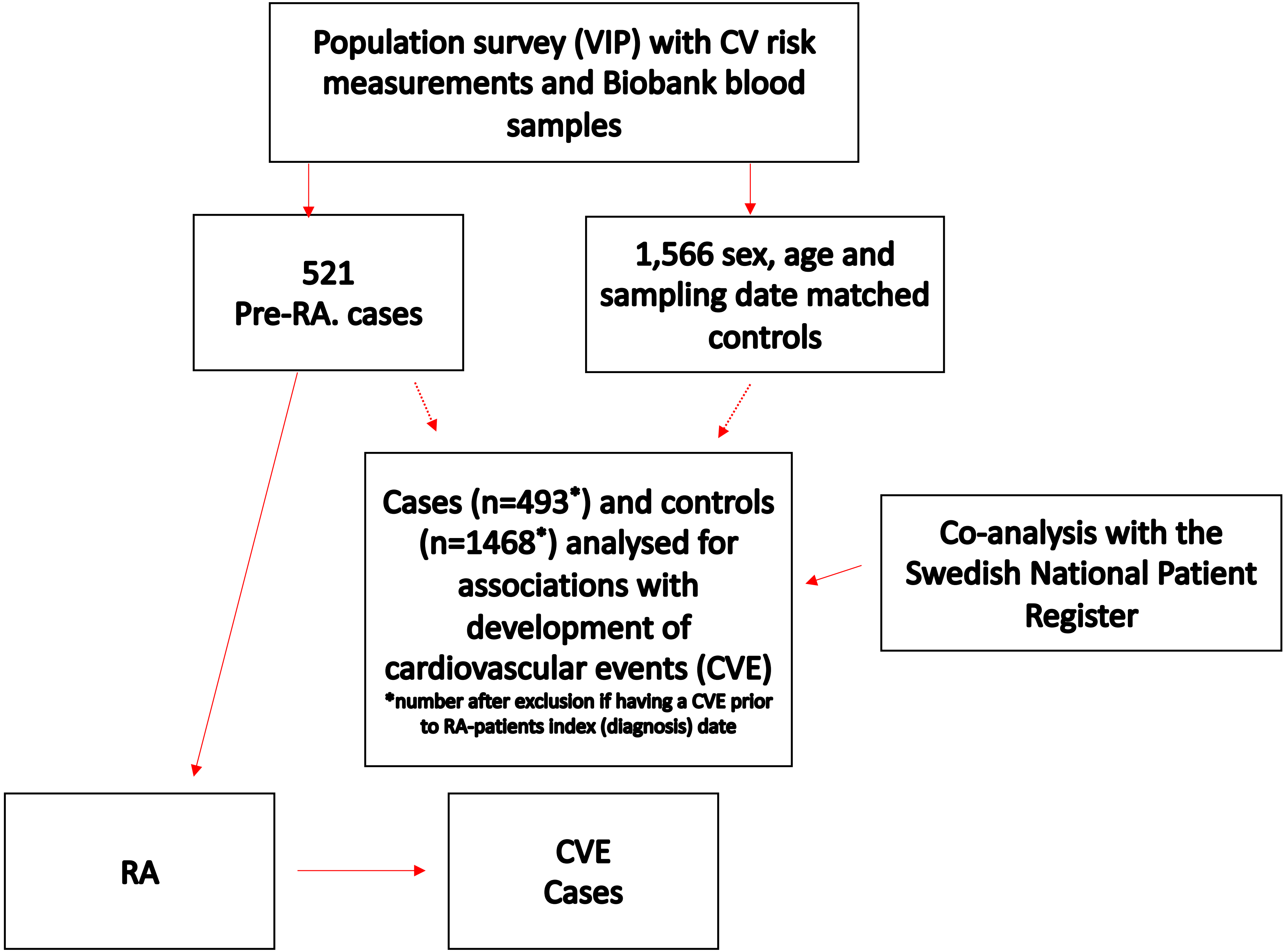

Supplementary Figure S1. Flowchart on the inclusion of pre-RA individuals and matched controls in the study.
