# Supplementary material for: Cardiovascular Risk Factors before Onset of Rheumatoid Arthritis Are Associated with Cardiovascular Events after Disease Onset: A Case–Control Study
Source: J Clin Med. 2022 Nov 3;11(21):6535. doi: 10.3390/jcm11216535 (PMC9658375; doi:10.3390/jcm11216535)
Supplement: Supplementary file 1 [file jcm-11-06535-s001.zip › Supplementary Table S1.pdf]

Supplementary Table S1. ICD[a]-10/-9 codes included as a CVE[b] due to CV comorbidity or mortality

ICD-10/9 code	
I20.0, I20.1, I20.8, I20.9, I24.9, I25.1 + Z95.1, or Z95.5	Unstable angina pectoris, acute ischemic heart disease, angina pectoris together with presence of aortocoronary bypass graft or presence of coronary angioplasty implant and graft
I21.0-I22.9, I24.9/410A,B,X,W	Acute myocardial infarction, subsequent ST elevation (STEMI) and non-ST elevation (NSTEMI) myocardial infarction,
I46.1	Cardiac arrest
I61.0, I63.0-9, I64.9, I65-I66.9/434A, B, X	Nontraumatic intracerebral hemorrhage, Cerebral infarction, Acute cerebrovascular disease, Occlusion and stenosis of precerebral arteries, Occlusion and stenosis of cerebral arteries
I71.1, I71.8	Aortic aneurysm (ruptured)
G45.9, G45.3/435X	Transient cerebral ischemic attack, amaurosis fugax

[a]ICD=International Statistical Classification of Diseases and Related Health Problems, [b]CVE=cardiovascular event
