# Supplementary material for: Cardiovascular Risk Factors before Onset of Rheumatoid Arthritis Are Associated with Cardiovascular Events after Disease Onset: A Case–Control Study
Source: J Clin Med. 2022 Nov 3;11(21):6535. doi: 10.3390/jcm11216535 (PMC9658375; doi:10.3390/jcm11216535)
Supplement: Supplementary file 1 [file jcm-11-06535-s001.zip › Supplementary Table S2.pdf]

Supplementary Table S2. Risk for CVE[a] after index date stratified on CVD[b] risk factors prior to RA onset.

**Hypertension[c]**	**Elevated ApoB:A1 ratio[d]**	**Current smoking**	**P-value**	**OR (95% CI)**
**Cases**				
-	-	-		Ref
-	+	+	0.010	4.74 (1.45-15.56)
+	+	-	$1.85x10^{-4}$	7.89 (2.67-23.29)
+	-	+	0.011	5.23 (1.46-18.77)
+	+	+	$2.90x10^{-5}$	12.07 (3.76-38.78)
**Controls**				
-	-	-		Ref
-	+	+	0.033	3.31 (1.10-9.90)
+	+	-	0.001	4.96 (1.97-12.47)
+	-	+	0.035	4.94 (1.12-21.75)
+	+	+	$2.0x10^{-6}$	10.87 (4.03-29.30)

Logistic regression analyses presented with odds ratios (ORs), 95% confidence intervals (CIs), adjusted for age and sex, [a]CVE=cardiovascular event, [b]CVD= cardiovascular disease [c]hypertension = systolic blood pressure≥140 and/or diastolic blood pressure ≥90 and/or medical treatment for hypertension, [d]elevated apolipoprotein (Apo)B/ApoA1-ratio = male sex ≥0.8, female sex ≥ 0.7
